# The German System

**Published:** 1911-07-15

**Authors:** 


### THE GERMAN SYSTEM.

BY OUR SPECIAL COMMISSIONER.

### IV.—STATE INSURANCE IN GERMANY. HOW IT AFFECTS HOSPITALS.

### SICKNESS INSURANCE.

The German system of compulsory insurance for workers in case of sickness as it at present exists is a modification of older systems which were altered and amended as details were found to be in need of modification. It is interesting to note that Bavaria possessed a "Nursing Insurance" scheme as early as 1856, and that tentative attempts to work on somewhat similar lines were initiated before Prince Bismarck introduced his first scheme which is the basis of the existing law. These beginnings, however, have at present merely an historical interest, and there is no need to discuss them in extenso. Nor is there any necessity to detail the provisions of the Act of 1889 or the earlier Act, since they have been abrogated and amended by the Act of 1892, which, in the amended form introduced in 1903, is now the law in Germany.

The *Krankenversicherungsgesetz* of April 10, 1892, consists of 87 paragraphs, and repeals the Acts of 1876 (*Reichsgesetzbl. S.* 134) (*Reichsgesetzbl. S.* 125), while it amplifies the Acts of 1883 (*Reichsgesetzbl. S.* 73) and of May 5, 1886. The first part deals with *Versicherungszwang*—that is the liability to insure. Under Section 1 such liability rests upon persons who in return for wages or pay are engaged in (*a*) mines, saltworks, manufactories, shafts, factories, railways or shipping, baggage conveyancing on quays or in building; (*b*) in trade, in manual labour, or in other manual occupations; (*c*) in offices of solicitors, notaries public, insurance societies, commercial undertakings, or the like; (*d*) in occupations in which apparatus worked by elementary power (wind, water, steam, gas, hot air, etc.) are used, provided such power is not used in a casual manner; (*e*) workers employed in post and telegraph service, provided they are not yet included under one or other of the aforementioned groups; (*f*) crews of ships. By a special sub-section apothecaries, assistants, and pupils are excluded from this section.

#### Who is a Worker.

By the term worker or labourer (*Arbeiter*) is understood one who is employed in the specific occupation for a term not less than a week. The Courts have held that a laundry-woman, employed in a charitable institution, is a worker in the sense of the Act. On the other hand, workers who receive no wages or pay (*i.e.* probationers in certain cases) are not included under this section. It has been held (Keidel, *Sämtliche Entscheidungen*, p. 65) that a female attendant at a hydrotherapeutic institute owned by a private individual is not a worker in the sense understood by the Act. Nurses, attendants, and servants generally are obliged to insure in private voluntary institutions. In general the rule is that if such persons are servants (*Gesinde*) they are not liable to insurance unless special provision has been made by *Landesgesetz.* How far the personnel of a public hospital can be classed as servants within the meaning of the Act depends on circumstances. The intention of the Legislature appears to have been to leave the question an open one. In Herr Secretary Dr. Delbruck's words, "the conditions under which the personnel in a hospital is employed are so diverse that it is impossible to group these persons under a single category."

Section 2 of the Act extends the obligations to agricultural labourers, artizans, servants, and other workers, but only when a community desires to apply this section within its own limits. Persons in an official position are not obliged to insure under the Act, nor is anyone who "exercises the calling of the medical profession." The Prussian Courts have held that employees in a private hospital are workers within the meaning of the Act, whether they act as servants of the owner of the institute or not, but that employees of a dentist are not wage-earners. Nurses and ward attendants employed at a public hospital are apparently excluded under the Act, though the matter has never been judicially decided.

Sections 3, 4, and 5 deal with conditions under which exemption from the obligation to insure may be granted, and with the definition of the word "sickness." In general the Courts have held, as the clause is vague in its wording, that an insured person is sick when and so long as he is in need of medical treatment. Wödtke defines sickness in the words of the Regierungskommissar during the third reading of the novello as "an abnormal disturbance of the state of health requiring medical treatment, medicine, or curative measures." Rosin defines it as "a definite abnormal pathological condition of the body through which is caused a temporary interference with the wage-earning capacity of the individual owing to the necessity for medical treatment." These definitions have given rise to much interesting and instructive discussion, and the decisions of the Courts, fully given by Keidel, are worth reading. Thus it has been held that drunkenness is not sickness within the meaning of the Act, while toothache, if it necessitates medical treatment, is accepted as sickness. Parturition is sickness within the meaning of the Act; convalescence is not.

#### Sick Pay and Sick Support.

Section 6 states that help during sickness may be given to the insured in the form of (*a*) from the beginning of the illness as free medical treatment, including spectacles, medicines, trusses, etc., and (*b*) in case of incapacity from the third day of the illness for each working day, sick pay at the rate of half the ordinary daily wage earned by the insured. Sick support ends at the latest thirteen weeks after the commencement of the illness; sick pay at the end of the thirteenth week after the first instalment has been paid. The insuring societies are bound to employ a qualified medical man; even when the insured person consents, they may not delegate the treatment to a non-qualified person. They are not obliged to pay for a consultation with a specialist, but may do so if they choose; nor need they pay for certain special methods of treatment unless these, in the opinion of the medical men, are imperatively necessary. Thus it has been held that the costs of special orthopædic treatment at a Zander institute are special costs under the Act, and need not be paid by the society. The filling of carious teeth has been held to be ordinary treatment, for the costs of which the society is responsible; so also the provision of special medicines, such as injections, sera, etc. When the insured person refuses to be treated by the club doctor, he has no right to demand payment of the costs of treatment, but may obtain the sick pay; if he goes to a doctor who has been a club doctor but who has been removed from that position, the sick pay may be refused by the society.

#### Hospital Treatment.

Of prime importance to hospital authorities is section 7, which runs as follows: Instead of the provisions prescribed under the preceding section, free treatment and nursing may be guaranteed to an insured person in a (recognised) hospital, under the following conditions:

(1) For those who are married, or who possess a household, or who are members of their family household, provided they give their consent; or without their consent for such persons when the nature of the sickness makes such demands for treatment and nursing as cannot be met by the patient's family, or when the illness is an infectious one, or when the patient has repeatedly acted against the advice of the club doctor, or when his condition is such that it demands imperatively prolonged and special care.

(2) For other sick insured persons unconditionally. During his sojourn in hospital the society is empowered to pay to the patient's family, under certain conditions, sick pay at a rate not exceeding the half of the ordinary rate. It has been held that this transfer of a patient by the club or society to a hospital is the privilege and not the duty of the society. Even when the doctor thinks it necessary that the patient should go to a hospital, the society may refuse to pay the costs of institutional treatment (decision of the Courts of Baden). When a patient refuses to go to a hospital the society may also refuse to pay further sick pay. Nor is a patient bound to remain in the hospital, though if he leaves the institution before the medical man thinks he is able to go, the society may refuse to pay his sick pay unless it can be shown that he has had good grounds for so acting. By the Act of 1903 the societies are empowered to make special rules for the admission of members into hospital.

Sections 8 to 19 deal with the composition and character of the Krankenkassen. Section 20 details the obligations of a Krankenkas towards its members. It must guarantee sick support and sick pay as prescribed under the sections already mentioned; maternity pay for at least four weeks, and burial pay (*Sterbegeld*) to an amount of at least twenty times the average ordinary daily wage of the member. Section 21 amplifies and extends these prescriptions, and provides for the free treatment, in case of sickness, of the family of members. Sections 22 to 45 deal with the conditions of membership and the composition of the boards of management, the regulation of the finances, and the corporate powers of the Krankenkassen. Section 46 gives the clubs the power to enter into contracts with medical men, hospitals, apothecaries, and druggists; to build and equip their own convalescent homes, convalescent institutes, and hospitals, and to carry out the specific provisions of their own statutes under the Act. While this section applies more particularly to combinations of societies, it has been held by the Courts that a single society is acting within its powers in building its own hospital.

The remaining clauses of the Act deal in detail with the various Krankenkassen (Orts- Betriebs- Bau- Hilf- Innung- Knappschafts- and provisional Kassen), and with the conditions under which members obtain their privileges and acquire their corresponding obligations. The nature of these Krankenkassen, although we have ventured to translate the term freely by "clubs," or "insuring societies," is such that it is unfair to compare them wholly with Friendly Societies or clubs in this country. For instance, the State prescribes certain forms of statutes; if the membership is below a certain number, the society automatically ceases to exist, but can be incorporated with another; societies may join forces and deal with a particular district (Orts-Krankenkassen). Annual meetings must be held at which full statements must be submitted to the members or to representatives apart from the directorate of the society, chosen by them; yearly registers and accounts must be forwarded to the State authorities, and provision is made for independent auditing. Under Section 23 authority is given to the Gemeindebehorde to establish a Kassenstatut for regulating the classes of the members, the nature and extent of the assistance to be granted to members, the subscriptions to be paid, the formation of the directorate, the calling of general or special meetings, and the alteration of such rules as are already made.

It will be seen that the Act does not fix any definite payment, in the way of subscriptions, by members. The novello to the Act of 1892 explains that the benefits under the Act are confined to the "Arbeiter," and the question is therefore merely what is understood by the term Arbeiter? The law has laid down that "By the term labourer is meant one who does not work on his own, but who is dependent for his income upon the payment granted to him by his employer." A workman who occupies himself with independent work—that is, on his own behalf—with a trade or occupation which falls under the categories mentioned as obligatory for insurance purposes, is therefore not obliged to insure; he is, as it were, his own employer. The question of wages has occupied the Courts in Germany very freely, and has led to certain more or less definite pronouncements, the sum total of which comes to this, that income derived from employment exceeding £120 a year (or even, as has been held in some cases, 2,000 marks) cannot be considered as wages in the meaning of the Act. For ordinary purposes it must therefore be taken that membership of a society is confined to those who are employees in the strict sense of the term, and whose wages inclusive of extras and overtime do not exceed £120 yearly. It has been provided, however, that other persons, whose yearly income does not exceed £100, may join the societies voluntarily. Every insured person who is not a member of a society, comes under the jurisdiction of the Gemeinde Krankenversicherung.

The contributions towards the insurance premiums are calculated on the following basis: the insured pays two-thirds, and the employer one-third of the weekly premium; the insured alone pays the entrance fee to a society. The amount to be paid in premiums is fixed by the society itself with the approval of the authorities.

### THE INVALIDITY INSURANCE ACT.

The clauses in the *Invalidenversicherungsgesetz*, of July 13, 1899, which affect more particularly the hospitals, are, in the main, an amplification and extension of similar clauses contained in the *Invaliditäts- und Alters- versicherungsgesetz* of June 22, 1889. Since the latter Act has entirely been replaced by the former, it is unnecessary here to detail the points of difference between the conceptions of hospital aid contained in each, and we may therefore confine our attention entirely to the Act of 1899.

The first clause of this enactment sets forth who are the persons included under the Act in the benefits to be derived from, and the obligations connected with, State insurance under compulsion.

Clause I.—According to the provisions laid down in this enactment the following are insured from the time they have completed their sixteenth year (*vom vollendeten sechzehnten Lebesjahre ab*:—

(1) Persons who are employed for wages or pay as labourers, assistants, helps, apprentices, or servants;

(2) Expert working men, artisans, technical workers, trade assistants and apprentices (with the exception of apprentices and pupils employed in apothecaries' shops), other employees whose employment in the capacity of servants form their chief occupation, as well as teachers and educators, together, in so far they receive pay or wages, provided their regular income from such employment does not exceed a sum of two thousand marks (£100) annually; as well as

(3) Those who are employed for pay or wages as members of the crew of any German vessel (section 2 of the Act of July 13, 1887. Reichsgesetzbl; section 329), or in any vessel or boat employed in river service within the boundaries of the Empire, captains of vessels, however, only on condition that their annual income from such employment does not exceed the sum of two thousand marks. The flying of the national flag in accordance with the law of 1888 *ipso facto* does not entitle the vessel to rank, for purposes of this Act, as a German vessel in the sense used in this Act.

In accordance with this clause the German authorities have from time to time given explanatory notes which more clearly differentiate between various classes of employees. It is at once evident that the clause dealing with *Versicherungspflichts* (that is, the duty or obligation upon the individual to insure himself in accordance with the provisions of the Act) is wider than the similar clause in the Act providing for sickness and accident insurance. The definitions are much less narrow, and a great deal is left for final interpretation. In consequence such interpretation has become necessary, and for a summary of these we are obliged to consider the decisions officially given on various test cases which from time to time have been brought up for adjudication. In the first place, it has been clearly laid down that the clause applies to both sexes, to men as well as to women; where a working woman marries, and, on giving up her ordinary employment, gets rid of the obligation to insure, such obligation again attaches when she resumes her ordinary work after marriage.

### The Limit of Income.

In deciding who are under the obligation to insure the question of wages or pay must be taken into account; this aspect of the matter is further considered in the following clauses of the Act, but so far as hospitals are concerned the following official notes are of great interest and importance.

Free gifts are not wages, and the service rendered in return for such gifts cannot be regarded as employment in the sense as defined in the Act. Private or public institutions for charitable objects, such as asylums, hospitals, idiot, blind, and insane asylums, pauper institutions, etc., are not on the same footing with other public or private firms or institutions where the employees receive definite wages. The employees in such charitable institutions may, however, receive definite wages or pay for definite services rendered, in which case they automatically come under the Act and become liable to pay their insurance contributions. Persons connected with a religious foundation, and exercising certain specific functions, also fall under the Act when they receive for their services definite and regular pay. The obligation to contribute commences from the first week in the seventeenth year of life. The official memorandum of December 19, 1899, deals with the personnel of hospitals and charitable institutions in the clearest and most definite manner, and the clauses relating to this matter may be summarised as follows :—

### Nurses and Hospital Staffs.

Persons who devote themselves to nursing and the care of the sick, practise in general an independent occupation, and are therefore not to be included under those who are provided for under the Act. Such independent employees are, for example, nurses who work on their own, masseurs and masseuses, midwives, voluntary nurses, and attendants who receive pay in proportion to the special work they do, and who are not in receipt of regular wages. Even when a midwife is employed by a corporation, and receives pay in proportion to the number of cases which she attends, it has been held that it is not obligatory on her to contribute towards her insurance. On the other hand, it has been decided that if a midwife undertakes to attend a woman as nurse for a period after the confinement, and is in receipt, while so doing, of a regular wage, she is bound to contribute. In so far as they render professional services, nurses and ward attendants are not included among the wage-earning class affected by the Act, and their employment in household duties, arising out of their professional engagements does not bring them under this category. When the nurse or attendant has not had a professional training, and does ordinary service in return for regular wage, he or she falls automatically under the wage-earning class, and it becomes obligatory on him or her to contribute. Similarly a nurse or attendant who is permanently employed as a servant of an institution, whether private or public, and is in receipt of a regular wage for such service, is compelled to contribute.

This interpretation makes it evident that under the Act wardmaids and male attendants as employed in English institutions are to be considered wage earners, while probationers and nurses exercising their practice are excluded, and sisters or head nurses who are in receipt of a regular wage may or may not be considered wage earners, according to whether or not they are paid for their professional services or employed as in an administrative or supervisory capacity.

### Some Test Cases.

In his interesting commentary on the decisions under the Act, R. Königl. Bezirksamtsassessor Keidel (*Sämtliche Entscheidungen des Reichsversicherungsamtes und des Reichsgerichts auf dem Gebiete der Invalidenversicherung*) discusses several instructive test cases. In one instance it was held that a communal midwife was not entitled to benefit under the Act since she was practising an independent occupation. Persons who made use of her services were deemed not to be "employers" in the sense of the Act. Nor was it held that she lost this independence when she accepted a position under a corporation which paid her so much for every confinement she attended, and which limited her work to a special district. A woman employed to lay out pauper corpses and who was in receipt of a regular wage for so doing, was, on the other hand, held to be a wage earner under the Act. With regard to nurses or ward attendants the authorities have clearly laid down that such persons are to be classed as wage-earners only when there is reason to believe that they are employed as labourers, and not as persons exercising an independent function. Voluntary nurses do not place their services at the disposal of their employers qua servants, but perform definite acts which require a certain amount of professional training and skill, which warrant the assumption that such persons are practising an independent occupation. The fact that they are under supervision and amenable to discipline, and that they may have to do servant's work, such as ordinary household work, does not militate against this, provided it can be shown that these persons are in possession of skilled knowledge. The position of mortuary attendants, assistants in the operating theatre, and helpers in the $x$-ray and in the special departments, is apparently analogous, in view of this decision, to that of the regularly trained nurse. The authorities have held, again, that a nurse employed to take care of poor patients in a particular district, in return for which she received free quarters and a regular wage, was to be regarded as a wage earner. This apparently contradictory decision was due to the fact that it had been shown that the nurse was not properly certificated, and that her duties were to perform menial work in the houses of the patients whom she visited. The decision has been rescinded since, and Keidel has no doubt that in this case even there is sufficient reason to believe that the services rendered were of a nature to be regarded as independent occupation. Similarly the Courts have held that the exercise of any service which requires special training and skill, and which is carried out independently, is not wage-earning service in the sense defined in the Act. An interesting corollary to these decisions is the verdict given with regard to patients in a hospital or infirmary who are engaged in certain occupations, and even the prisoners in a gaol who are paid a certain fixed sum for the work they perform. Are such persons wage earners? Is a hospital patient during his convalescence, or a sanatorium patient engaged in outdoor work, a wage-earner in the sense defined in the Act? The question has largely centred not round the Invalidity Act, but round the Old Age Pension Act. In cases where such persons have claimed to be entitled to old-age pensions on the ground that they have been wage earners, the Courts have held that it must be proved that they were in receipt of regular wages, though the fact that they were in receipt of free board and lodging has been taken into consideration in adjudicating the amount of wage earned. Where the wages have been regular, and given in return for services rendered, the claim has been upheld.

The position of a nurse who is in receipt of a regular wage, and is employed by a special institution or association, society, or order, is apparently different from that of one who is employed in a hospital, or who is a member of a definite nursing corporation or sisterhood. The latter has no claim to the benefits under the Act, and is not obliged to contribute. The former, however, is a wage earner within the meaning of the Act. It must be remembered that the pay given to nurses in German hospitals is lower than that granted in English institutions, and that therefore such pay, especially when the nurse is trained, and there is therefore no question about her exercising professional functions, may be looked upon as pocket money rather than as wages. The Courts have held that a nurse not a member of a regular sisterhood, who had been employed at a yearly wage of 300 marks by a nursing association, was entitled to benefit under the Act.

### Voluntary Insurance.

Section 3 of the Act deals with the definition of wages, and defines what equivalents may be accepted as such. Gratuities and sums given as pocket money are not held to be wages within the meaning of the Act. Equivalents in the shape of instruction given, education, free board and lodging, free lodging or board alone, and so on are discussed.

Section 5 deals further with those who are or are not benefited by the Act, with special reference to teachers in public schools or educational institutions. This section has some interest for hospitals with medical schools attached. It seems that a house surgeon, or an unqualified student, working in the wards would come under the class of wage earners, since it has been held that pupil teachers are wage earners under the Act. It is more likely, however, that such persons would be looked upon as falling under the category of partly skilled male nurses, to whom the decisions already quoted apply.

Section 14 deals with voluntary insurance. Voluntary insurers must be below forty years of age, and in receipt of wages or salary not below two thousand marks, and not exceeding three thousand marks per year. Persons compulsorily insured under Section 1 may, when through being in receipt of higher wages or some other cause which renders them ineligible to continue as first-class insurers they are debarred from the benefits of the Act, insure themselves voluntarily under this section.

Section 16 defines the conditions under which a person loses his or her right to insurance—for instance, if he or she is in receipt of a pension through being permanently disabled, or in receipt of an old age pension.

Section 17 expressly states that invalidity benefit does not accrue when the insured person has deliberately produced the disability; the benefits under the Act may be wholly or partly lost if the insured has occasioned the disablement by committing some act or acts which may be considered as crimes or misdemeanours; in the latter case the authorities may, however, pay part of or the whole sum to the family or relatives of the insured.

### Disablement.

The important section 18 deals wholly with the question of disablement. It provides that, in case an insured person is disabled from work, through sickness or other cause, the insuring society or club may provide for suitable treatment. The insuring society may insure such treatment by causing the patient to be admitted to a hospital. If the insured person be married, or if he has a family, or is a member of a household, his sanction must be obtained before he can be taken to a hospital. If the treatment is carried out on behalf of the insuring society, whether at a hospital or elsewhere, that society is responsible for the costs of such treatment, which must guarantee the hospital the amount. Keidel instances the following case: An insurance institution caused an insured person, who desired his invalidity money, but whose case was declared by the doctors to be curable, to be taken to a hospital in terms of this section. Such treatment occasioned a marked improvement in the patient's condition, and the insuring institution declared that there was no reason to continue payment, since there was no question of permanent disablement. It declared, however, its willingness to pay for a further course of hospital treatment. The arbitrators accordingly decreed that the insuring institution should pay the full costs of the treatment.

Keidel, in the volume already referred to, gives several instructive cases in which this section has been subjected to judicial amplification. From these it appears that the insuring society is not responsible for the costs of burial in case the patient dies while in hospital.

Sections 19 to 22 deal with the time-limits and the obligations of the sick clubs "Krankenkassen," with regard to the insured. Section 25 further specifies that the Versicherungsanstalt may, at the request of the insured, cause the latter to be taken into a hospital at the expense of the insuring Anstalt, and in lieu of the weekly payment to such insured.

Section 30 deals with payments to the insured, and states how they are to be reckoned. Section 31 (proof of illness) and section 32 (amount to be paid) are to be taken in conjunction with sections 33, 34, 35, 36, 37, 38, 39, 40, and 41, which deal with the financial aspect.

For the purposes of the Act the insured are divided into five classes: Class I. (in receipt of wages not exceeding 350 marks per year) pays a weekly premium of 14 pfennig; Class II. (from 350 to 550 marks) pays 20 pfennigs per week; Class III. (550 to 850 marks) pays 24 pfennig; Class IV. (850 to 1,150 marks) pays 30 pfennigs; and

Class V. (above 1,150 marks) pays 36 pfennigs per week. The amounts due to the insured in case of invalidity are reckoned according to the classes, and according to the length of time the insured has paid his premiums. Section 27 defined the relative amounts to be contributed by the wage earner and the employer, both of whom contribute equal shares, while the State gives a subsidy in proportion to the amount to be paid out, at the rate of 50 marks yearly for each payment. The Grundbetrag, or fixed amount of each policy varies in each class, from 60 marks in Class I. to 100 marks in Class V. To this is added the Steigerungsbetrag calculated at the rate of from 3 pfennigs per week in the first, to 12 pfennigs per week in the fifth class. The payment starts from the day on which disablement is certified. The further sections of the Act are of relatively little importance to hospitals. They deal with the practical details of the scheme, define the constitution of the various bodies involved, and regulate the minor details of working.

Accident Insurance.

The Accident Insurance Law is of relatively little importance to hospitals. By the official memorandum of the Versicherungsamt (85 sect. 366) it is laid down that persons employed in a hospital as mechanics, lift attendants, bath, laboratory, laundry, bakery attendants, and in any occupation which is mechanical, are to be insured. It has been proposed that the hospitals should join together and found a separate insurance society under the Act; many institutions have already privately insured their whole staff against accident.

